# Effectiveness of an improved fall risk assessment form combined with obstacle physical activity testing in preventing falls in older adults hospitalized patients

**DOI:** 10.3389/fpubh.2025.1601666

**Published:** 2025-10-28

**Authors:** Xuan Wang, Wei Li, Meijie Zheng, Chengfei Li, Ruijing Liang, Suchan Yao, Xiaoli Liu, Xiaosong Zhang, Xiaomin Di, Yang Lu

**Affiliations:** ^1^Department of Geriatric Rehabilitation, The First Hospital of Hebei Medical University, Shijiazhuang, Hebei, China; ^2^Department of Senile Cardiovascular Disease, Hebei General Hospital, Shijiazhuang, Hebei, China; ^3^Department of Digestive System, Hebei General Hospital, Shijiazhuang, Hebei, China; ^4^Department of Interventional Therapy, The First Hospital of Hebei Medical University, Shijiazhuang, Hebei, China; ^5^Department of Geriatric, The First Hospital of Hebei Medical University, Shijiazhuang, Hebei, China; ^6^Department of Urology, The First Hospital of Hebei Medical University, Shijiazhuang, Hebei, China; ^7^Shijiazhuang City Emergency Rescue Center, Shijiazhuang, Hebei, China; ^8^Department of Hematology, The First Hospital of Hebei Medical University, Shijiazhuang, Hebei, China; ^9^Department of Spinal Surgery, The First Hospital of Hebei Medical University, Shijiazhuang, Hebei, China

**Keywords:** older adults hospitalized patients, falls, obstacle physical activity ability test, fall risk assessment, fall prevention, patient safety

## Abstract

**Objective:**

This study aimed to evaluate the effectiveness of personalized preventive interventions guided by an improved Risk Assessment Form and an obstacle physical activity test in preventing falls among older adults hospitalized patients.

**Method:**

A single-center, randomized controlled trial was conducted with 320 older adults hospitalized patients (mean age 76.4 ± 6.8 years), who were allocated to either an experimental group (*n* = 160) or a control group (*n* = 160). The experimental group received a comprehensive fall risk assessment using an improved form and an obstacle activity test, which subsequently guided personalized prevention measures. The control group was assessed using traditional hospital fall risk screening methods and received standard fall prevention care. The primary outcome was the incidence of falls. Secondary outcomes included injury severity, nursing satisfaction, patient compliance, physical activity improvement, and quality of life. Key areas for process improvement were identified using Failure Mode and Effects Analysis (FMEA).

**Result:**

The experimental group had a significantly lower fall incidence (8.13%) compared to the control group (28.13%). The experimental group also experienced a lower severity of injuries, with a higher proportion of soft tissue injuries and a lower proportion of fractures. Nursing satisfaction, patient compliance rates, physical activity improvement, and quality of life scores were all significantly higher in the experimental group compared to the control group. FMEA identified that failure to implement preventive measures consistently was the highest-risk failure mode in the fall prevention process.

**Conclusion:**

The application of personalized fall prevention strategies guided by a comprehensive assessment that combines a multidimensional risk form with a dynamic obstacle physical activity test is effective in reducing falls and injury severity among older adults hospitalized patients. This approach also enhances patient satisfaction, compliance, and quality of life, and is recommended for broader implementation in inpatient settings.

## 1 Background

In the field of medical care, the safety of older adults inpatients remains a paramount concern. Falls, as a common adverse event, not only cause physical and psychological harm to patients but also impose significant burdens on medical institutions, including increased length of stay, higher healthcare costs, and potential legal liabilities (1–3). According to statistics, falling is a leading cause of injury-related death globally, and among older adults, it is the primary cause of non-fatal injuries (4, 5). About one-third of community-dwelling older adults fall each year, with the incidence rate rising with age and frailty (6–8). In individuals over 80, the annual fall rate can reach up to 50% (9). Such falls frequently lead to severe complications, including traumatic brain injuries, soft tissue damage, fractures, and dislocations, all of which profoundly affect patients’ quality of life and functional independence (10). Despite numerous existing fall risk assessment tools, many have limitations in predicting falls in diverse clinical settings, particularly in capturing dynamic risk factors related to interactions with the environment (11, 12). Recent systematic reviews continue to emphasize the need for improved assessment strategies that incorporate functional and environmental interactions to enhance predictive accuracy and guide targeted interventions (13, 14).

The etiology of falls in older inpatients is multifactorial, encompassing intrinsic physiological factors (e.g., muscle weakness, impaired balance, osteoporosis), cognitive impairments (e.g., delirium, dementia, poor judgment), and extrinsic environmental hazards (e.g., slippery floors, poor lighting, obstacles) (15–17). Additionally, nursing management factors cannot be overlooked, such as communication gaps, inconsistent application of protocols, and challenges in predicting fall risk accurately (7, 18, 19). Effective fall prevention requires a multifaceted approach that goes beyond static risk factor identification to include dynamic assessments and tailored interventions, as demonstrated by Bhasin et al. (20) in their randomized trial of a standardized multifactorial strategy incorporating motivational interviewing, individualized care planning, and follow-up to address specific risk profiles. This approach is reinforced by systematic reviews confirming that multifactorial interventions must be customized to individual risk factors rather than applying uniform solutions (21), and aligns with clinical guidelines recommending prompt, targeted interventions based on comprehensive assessments of modifiable risks such as gait, balance, medication use, and environmental hazards (22). Consequently, it has become an urgent issue to implement and validate more effective preventive strategies to reduce the incidence of falls among older adults inpatients.

To address this gap, this study developed and evaluated an intervention package centered on an improved “Risk Assessment Form for Inpatient Falls and Bed Falls” (see Supplementary Table S1) combined with a novel obstacle physical activity test. While traditional screening tools like the Morse Fall Scale or Hendrich II Fall Risk Model are widely used, they primarily rely on a checklist of static risk factors (e.g., history of falls, secondary diagnosis) (23). Even functional assessments like the Timed Up and Go (TUG) test, while valuable, may not fully capture a patient’s ability to navigate a cluttered or unpredictable environment. Our approach sought to bridge this gap by combining a comprehensive, multidimensional assessment with a functional test that directly simulates environmental challenges. The purpose of this study is to explore the impact of personalized interventions guided by this improved risk assessment form, combined with obstacle physical activity testing, on fall incidence, injury severity, and other key patient-centered outcomes, including satisfaction and quality of life, for older inpatients through a randomized controlled trial.

## 2 Materials and methods

### 2.1 Study design and population

This study was a single-center, pragmatic, randomized controlled trial conducted at The First Hospital of Hebei Medical University, a tertiary care teaching hospital in China. The study protocol was approved by the Ethics Committee of The First Hospital of Hebei Medical University and was performed in accordance with the Declaration of Helsinki. Written informed consent was obtained from all participants or their legal guardians.

This study was registered with ClinicalTrials.gov (Identifier: NCT07126925), the trial protocol is publicly accessible through the U. S. National Institutes of Health clinical trials registry (https://register.clinicaltrials.gov/).

Participants were recruited between January 2023 and December 2023 from the geriatric, orthopedic, and general internal medicine wards. Patients were screened for eligibility by the nursing staff upon admission.

#### 2.1.1 Sample size calculation

The sample size was determined based on the primary outcome: fall incidence. Based on institutional data and published literature, we anticipated a fall rate of approximately 28% in the control group. We aimed to detect a clinically significant reduction to 8% in the experimental group. Using an alpha of 0.05 (two-tailed) and a power of 90%, a sample size calculation for two independent proportions indicated that 121 patients per group would be required. To account for potential dropouts and to ensure sufficient power for secondary outcome analyses, we targeted a larger sample size of 160 patients per group.

#### 2.1.2 Eligibility criteria

Inclusion criteria:

- Inpatients aged 65 and above.
- Identified as being at risk of falling upon admission screening (defined as having at least one of the following: history of a fall in the last 6 months, use of a walking aid, or observed gait/balance instability).
- Able to provide informed consent or have a legal guardian provide consent.

Exclusion criteria:

- Acutely life-threatening conditions or severe cardiorespiratory instability that would preclude any mobility testing.
- History of a severe fall-related injury (defined as a fracture or head injury requiring hospitalization) in the past 6 months that currently limits mobility assessment.
- Non-ambulatory or bed-bound patients.
- Patients with severe dementia (e.g., Mini-Mental State Examination score < 10) or diagnosed psychiatric conditions (e.g., psychosis, severe agitation) that would prevent cooperation.
- Expected hospital stay of less than 48 h.

#### 2.1.3 Randomization and blinding

Eligible and consenting patients were randomly assigned in a 1:1 ratio to either the experimental or control group. The randomization sequence was generated by a statistician not involved in patient recruitment using a computer-based random number generator with permuted blocks of varying sizes (4, 6, and 8) to ensure balanced allocation. Assignments were concealed in sequentially numbered, sealed, opaque envelopes. A designated research nurse, not involved in patient assessment or care, opened the next envelope in sequence to reveal group allocation after a patient was enrolled. Due to the nature of the intervention, blinding of patients and the nursing staff delivering the care was not feasible. However, the outcome assessors responsible for collecting data on falls, injuries, and QoL were blinded to group allocation wherever possible.

### 2.2 Interventions

#### 2.2.1 Control group

Patients in the control group received the hospital’s standard of care for fall prevention. This included universal fall precautions for all older patients (e.g., ensuring a clutter-free environment, providing non-slip footwear, ensuring call bell is within reach) and a standard risk assessment using the hospital’s existing protocol, which is a checklist based on static factors like age, fall history, and medication use. Interventions were standard and not explicitly tailored to dynamic functional deficits.

#### 2.2.2 Experimental group

In addition to the universal fall precautions, patients in the experimental group underwent a comprehensive assessment using two specific tools: the “Improved Risk Assessment Form for Inpatient Falls” (Supplementary Table S1) and the “Obstacle Physical Activity Ability Test.” The results of this detailed assessment were used by the nursing and physical therapy team to develop a personalized fall prevention plan. Examples of personalized interventions included targeted balance and strength exercises, specific training on navigating obstacles, environmental modifications in the patient’s room (e.g., adjusting furniture), and enhanced patient/family education focused on specific identified risk behaviors.

### 2.3 Assessment tools and procedures

#### 2.3.1 Improved risk assessment form for inpatient falls

This form (Supplementary Table S1) was an enhanced version of the hospital’s standard tool, developed by a multidisciplinary team. It expanded on traditional static risk factors to include more detailed modules on physical activity ability (e.g., stability during transfers), balance function (static and dynamic tests), and cognitive status related to safety awareness. The total score is a simple summation of the points from each item, with a score ≥ 8 indicating high risk. The form was administered by a trained research nurse within 24 h of admission.

#### 2.3.2 Obstacle physical activity ability test

This test was designed to assess a patient’s functional mobility and balance in response to simulated environmental challenges. The test was a timed circuit that included: (1) rising from a chair without using arms; (2) walking 3 meters to a set of two low obstacles (15 cm high foam blocks) placed 1 meter apart; (3) walking around the obstacles; (4) navigating through a narrow passage (70 cm wide between two chairs); (5) walking an additional 3 meters over a textured mat to simulate uneven ground; and (6) returning to the chair and sitting down. Time to complete, number of stumbles or stability losses (contact with a wall/chair for support), and gait deviations were recorded. This test was administered within 48 h of admission.

#### 2.3.3 Failure mode and effects analysis (FMEA)

The risk value, or Risk Priority Number (RPN), was calculated by considering both the severity (S) and frequency (O) scores of potential failures in the fall prevention process. A consensus panel of 10 senior clinical staff (geriatricians, nurse managers, physical therapists) rated each mode based on their expert opinion and institutional incident data, using an internal scale. The scores in Table 1 represent the average rating from this panel. The risk value was determined using the formula: Risk Value = Severity (S) × Frequency (O). This system was used as a quality improvement tool to prioritize areas for intervention, with higher scores indicating greater risk.

**Table 1:** Risk assessment of potential failure modes in the fall prevention process.

Failure mode	*Severity (S) (Average score)	*Frequency (O) (Average score)	Risk value (S × O)
Inaccurate initial risk assessment	3.37	2.53	8.53
Preventive measures are not implemented	4.22	3.25	13.72
Patients do not comply with measures	3.54	2.78	9.84
Environmental obstacles are not cleared	4.38	2.54	11.13
Slippery ground not addressed in time	3.57	3.52	12.57
Risk assessment form is incomplete	2.14	1.56	3.34
Insufficient training of nursing staff	3.27	1.13	3.70
Insufficient lighting at night	3.55	1.55	5.50
Emergency call system is slow to respond	2.53	2.26	5.72
Physical activity ability test is inaccurate	4.12	2.22	9.15

*Severity (S) and Frequency (O) scores represent average ratings from a consensus panel of 10 senior clinical staff. The values are used for internal quality improvement prioritization rather than as clinical diagnostic scores.

#### 2.3.4 Outcome measures

Data on outcomes were collected throughout each patient’s hospital stay by trained outcome assessors who were blinded to group allocation.

- Primary outcome (Fall Incidence): the number of falls per patient was recorded through the hospital’s incident reporting system, patient self-report, and daily nursing checks. A fall was defined as an event which results in a person coming to rest inadvertently on the ground or floor or other lower level.
- Injury severity: the severity of any fall-related injury was classified by a physician as: no injury, minor injury (e.g., bruises, abrasions), or major injury (e.g., fracture, head injury with loss of consciousness, laceration requiring sutures).
- Nursing satisfaction: assessed at discharge using a 5-item institutional survey where patients rated their satisfaction with nursing communication, responsiveness, and fall prevention education on a 10-point scale.
- Patient Compliance: assessed weekly via a 10-item observational checklist of prescribed precautions (e.g., call bell in reach, bed in low position, adherence to activity restrictions). Compliance was categorized based on the percentage of items adhered to.
- Physical activity improvement: categorized by the treating physical therapist at discharge as “Improved markedly,” “Raised (improved),” “Uniformity (no change),” or “Reduced,” based on a clinical judgment of change in performance on the obstacle test and general mobility from admission to discharge.
- Quality of Life (QoL): assessed at admission and discharge using a structured, non-validated questionnaire (Supplementary File S2) covering physical, psychological, and social domains. In addition to categorical improvement, the mean change in a 10-point global QoL rating was calculated.

### 2.4 Statistical analysis

Data analysis was performed using statistical software SPSS 26.0. Baseline characteristics of the two groups were compared using independent samples t-tests for continuous variables and chi-square (*χ*^2^) tests for categorical variables. The primary outcome, fall incidence (proportion of patients who fell), was compared using a *χ*^2^ test. Injury severity, compliance, and improvements in physical activity and QoL were also compared using *χ*^2^ tests. Mean nursing satisfaction scores and QoL score changes were compared using independent samples t-tests. Exact *p*-values were reported for all tests. A *p*-value of less than 0.05 was considered statistically significant. Given the pre-specified primary and key secondary outcomes, no formal correction for multiple comparisons was applied.

## 3 Results

### 3.1 Participant characteristics

**Figure 1:**
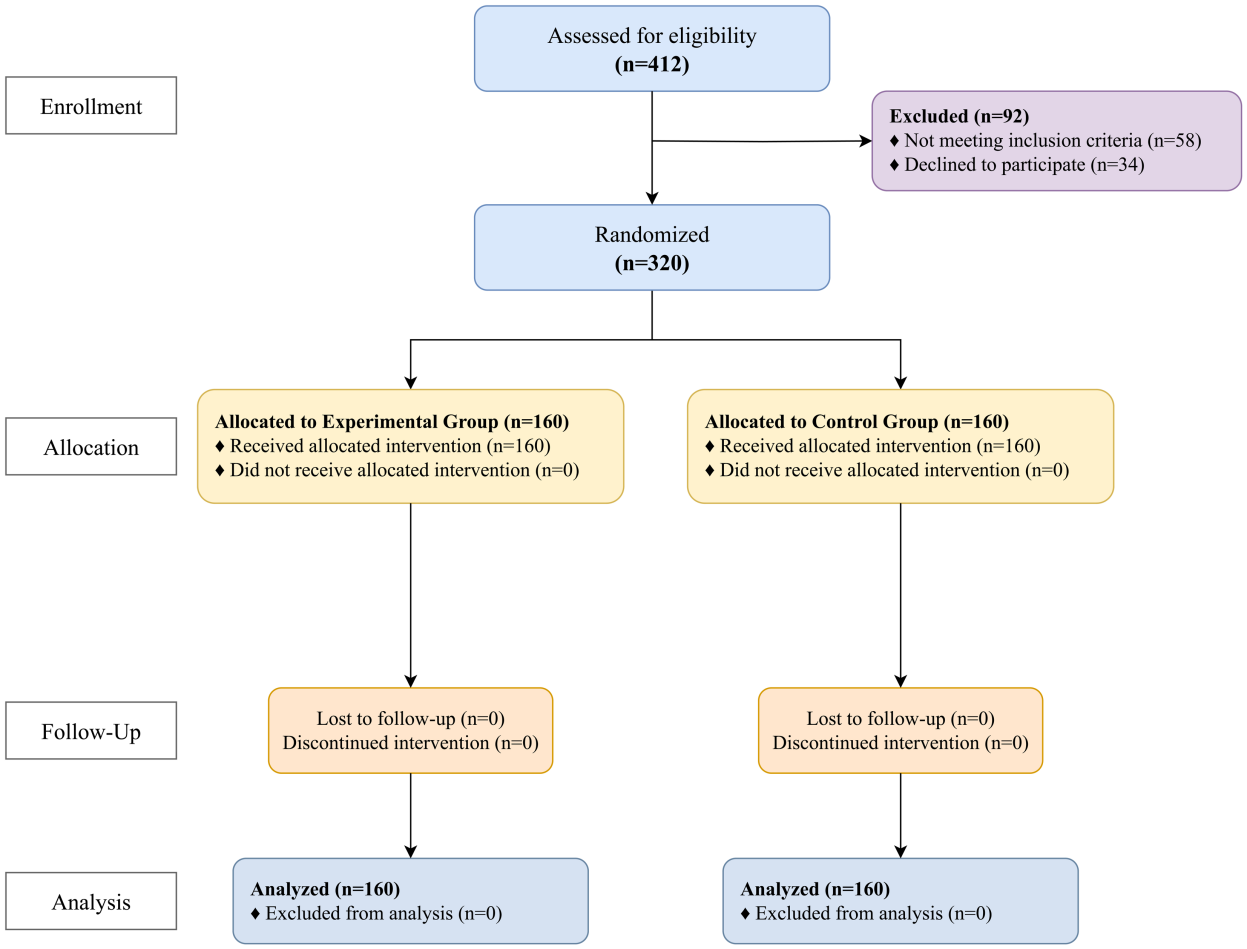
CONSORT flow diagram of participant recruitment and allocation.

**Figure 2:**
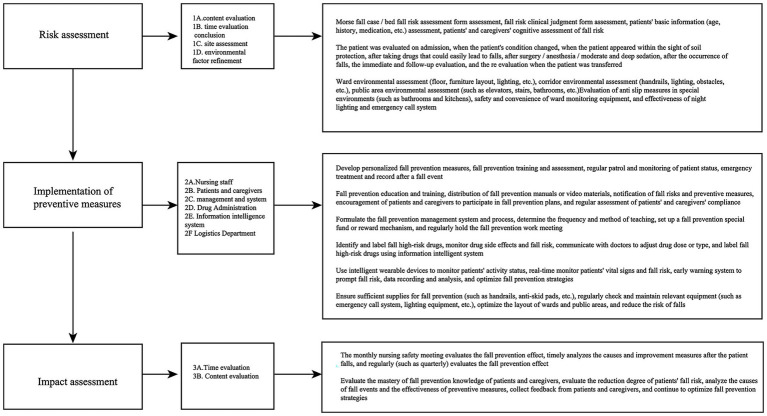
Flowchart of fall prevention for older adult inpatients.

A total of 320 patients were enrolled and randomized, with 160 in each group. All participants completed the study, and their data were included in the analysis (Figure 1). The baseline demographic and clinical characteristics of the two groups were well-balanced, with no statistically significant differences observed in age, sex, key comorbidities, or baseline mobility scores (Table 2). The mean age of the total sample was 76.4 ± 6.8 years, and 54.7% were female (Figure 2).

**Table 2:** Baseline demographic and clinical characteristics of participants.

Characteristic	Experimental group (*n* = 160)	Control group (*n* = 160)	Statistic	*p*-value
Age (years), mean ± SD	76.2 ± 6.9	76.6 ± 6.7	*t* = −0.498	0.619
Female, *n* (%)	89 (55.6%)	86 (53.8%)	*χ*^2^ = 0.091	0.763
Hypertension, *n* (%)	101 (63.1%)	105 (65.6%)	*χ*^2^ = 0.229	0.632
Diabetes Mellitus, *n* (%)	52 (32.5%)	48 (30.0%)	*χ*^2^ = 0.223	0.637
Osteoporosis, *n* (%)	78 (48.8%)	71 (44.4%)	*χ*^2^ = 0.692	0.405
History of Fall (past year), *n* (%)	45 (28.1%)	49 (30.6%)	*χ*^2^ = 0.252	0.616
Baseline Mobility Score (1–5), mean ± SD*	3.1 ± 0.9	3.2 ± 1.0	*t* = −0.875	0.382

*Baseline mobility score assessed by nursing upon admission on a 5-point scale (1 = fully dependent, 5 = fully independent).

### 3.2 Comparison of fall incidence

**Figure 3:**
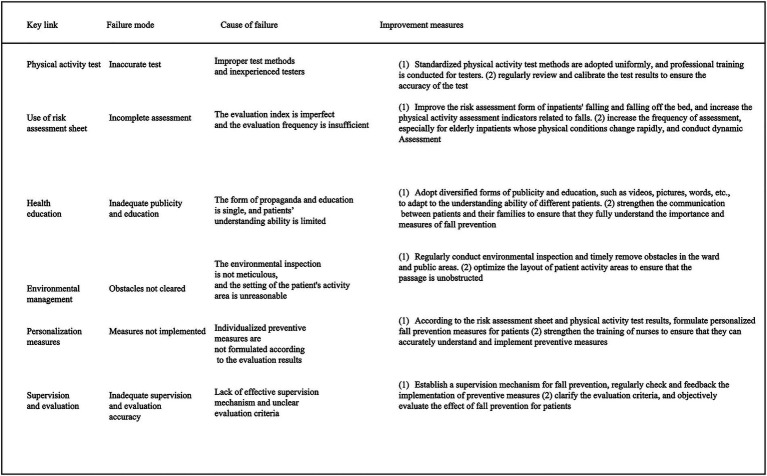
Table of key links, failure modes, failure reasons and improvement measures of fall prevention process for older adult inpatients.

The incidence of patients experiencing one or more falls in the experimental group (8.13%, 13 of 160) was significantly lower than that in the control group (28.13%, 45 of 160), and this difference was statistically significant (*χ*^2^(1) = 22.595, *p* < 0.001) (Table 3; Figure 3).

**Table 3:** Comparison of fall incidence.

Group	Number of falls	Incidence of falls	Statistic (*χ*^2^)	*p*-value
Control group (*n* = 160)	45	28.13%	22.60	<0.001
Experimental group (*n* = 160)	13	8.13%		

### 3.3 Comparison of fall injury sites

Among patients who fell, a statistical comparison of the injury sites revealed that the proportion of patients with soft tissue injuries in the experimental group (61.54%) was significantly higher than in the control group (22.22%) (*χ*^2^ = 6.887, *p* = 0.009). Conversely, the proportion of patients with fractures was lower in the experimental group (23.08%) than in the control group (44.44%) (*χ*^2^ = 4.018, *p* = 0.045) (Table 4).

**Table 4:** Comparison of fall injury sites.

Injury type	Experimental group (*n* = 13 falls)	Control group (*n* = 45 falls)	Statistic (*χ*^2^)	*p*-value
Soft tissue injury, *n* (%)	8 (61.54%)	10 (22.22%)	6.89	0.009
Fracture, *n* (%)	3 (23.08%)	20 (44.44%)	4.02	0.045
Head injury, *n* (%)	1 (7.69%)	5 (11.11%)	0.21	0.651
Joint dislocation, *n* (%)	1 (7.69%)	5 (11.11%)	0.21	0.651
Other, *n* (%)	0 (0.00%)	5 (11.11%)	1.54	0.214

### 3.4 Comparison of nursing satisfaction

Regarding nursing satisfaction, the experimental group showed a significantly higher number of “very satisfied” patients (*χ*^2^ = 15.233, *p* < 0.001) and a higher average satisfaction score (8.90 ± 0.22) compared to the control group (7.50 ± 0.17) (*t* = 56.052, *p* < 0.001). Additionally, the percentage of dissatisfied patients in the experimental group (4.38%) was significantly lower than in the control group (19.38%) (*χ*^2^ = 18.600, *p* < 0.001) (Table 5).

**Table 5:** Comparison of nursing satisfaction.

Group	Very satisfied, *n* (%)	Satisfied, *n* (%)	Dissatisfied, *n* (%)	Average satisfaction (out of 10), mean ± SD
Control group (*n* = 160)	63 (39.38%)	66 (41.25%)	31 (19.38%)	7.50 ± 0.17
Experimental group (*n* = 160)	97 (60.63%)	56 (35.00%)	7 (4.38%)	8.90 ± 0.22
Statistic	χ2 = 15.233	χ2 = 0.918	χ2 = 18.600	t = 56.052
*p*-value	<0.001	0.338	<0.001	<0.001

### 3.5 Comparison of patient compliance

In terms of patient compliance, the rate of “complete compliance” in the experimental group (63.75%) was significantly higher than in the control group (44.38%) (*χ*^2^ = 12.444, *p* < 0.001). Furthermore, the percentage of patients who showed “disobedience” was significantly lower in the experimental group (4.38%) compared to the control group (19.38%) (*χ*^2^ = 18.600, *p* < 0.001). The overall compliance rate (complete + partial) was 95.63% in the experimental group versus 80.63% in the control group (*χ*^2^ = 20.585, *p* < 0.001) (Table 6).

**Table 6:** Comparison of patient compliance with preventive measures.

Group	Complete compliance, *n* (%)	Partial compliance, *n* (%)	Disobedience, *n* (%)	Overall compliance rate (%), *n* (%)
Control group (*n* = 160)	71 (44.38%)	58 (36.25%)	31 (19.38%)	129 (80.63%)
Experimental group (*n* = 160)	102 (63.75%)	51 (31.88%)	7 (4.38%)	153 (95.63%)
Statistic (*χ*^2^)	12.444	0.610	18.600	20.585
*p*-value	<0.001	0.435	<0.001	<0.001

### 3.6 Comparison of the improvement of physical activity

The improvement in physical activity ability was significantly greater in the experimental group. The overall improvement rate (90.01%) was significantly higher than that in the control group (60.01%) (*χ*^2^ = 36.036, *p* < 0.001). Specifically, more patients in the experimental group were rated as “improve markedly” (*χ*^2^ = 14.801, *p* < 0.001) (Table 7).

**Table 7:** Comparison of improvement in physical activity ability.

Group	Improve markedly, *n* (%)	Raised, *n* (%)	Uniformity, *n* (%)*	Reduced, *n* (%)	Improvement rate (%), *n* (%)
Control group (*n* = 160)	33 (20.63%)	63 (39.38%)	47 (29.38%)	17 (10.63%)	96 (60.01%)
Experimental group (*n* = 160)	65 (40.63%)	79 (49.38%)	11 (6.88%)	5 (3.13%)	144 (90.01%)
Statistic (*χ*^2^)	14.801	3.165	24.018	6.660	36.036
*p*-value	<0.001	0.075	<0.001	0.010	<0.001

*“Uniformity” indicates no significant change or a stable condition in physical activity ability from admission to discharge.

### 3.7 Comparison of quality of life

Changes in quality of life were also significantly more favorable in the experimental group. The overall QoL improvement rate in the experimental group (93.13%) was significantly higher than in the control group (65.01%) (*χ*^2^ = 35.061, *p* < 0.001). Similarly, the mean improvement in the 10-point QoL score from admission to discharge was significantly greater in the experimental group (2.5 ± 1.1 points) compared to the control group (0.8 ± 1.3 points) (*t* (318) = 13.45, *p* < 0.001) (Table 8).

**Table 8:** Comparison of changes in quality of life.

Group	Improve markedly, *n* (%)	Raised, *n* (%)	Uniformity, *n* (%)*	Reduced, *n* (%)	Improvement rate (%), *n* (%)
Control group (*n* = 160)	39 (24.38%)	65 (40.63%)	39 (24.38%)	17 (10.63%)	104 (65.01%)
Experimental group (*n* = 160)	73 (45.63%)	76 (47.50%)	7 (4.38%)	4 (2.50%)	149 (93.13%)
Statistic (*χ*^2^)	16.580	1.244	22.094	8.294	35.061
*p*-value	<0.001	0.265	<0.001	0.004	<0.001

*“Uniformity” indicates no significant change or a stable condition in quality of life from admission to discharge.

### 3.8 Failure modes in the fall prevention process

In the analysis of failure modes, the failure of preventive measures to be properly implemented or followed had the highest risk value (13.72), followed by the failure to address wet ground hazards in a timely manner (12.57). This indicates that failures in process execution posed a greater risk than failures in initial assessment. In comparison, the failure due to incomplete information in the risk assessment form had the lowest risk value (3.34) (Table 1).

## 4 Discussion

In this study, the practical application of personalized fall prevention strategies guided by the improved Risk Assessment Form and the obstacle physical activity test was explored in depth. The results indicate that this comprehensive assessment and intervention approach has clear advantages in reducing the incidence of falls, decreasing the severity of fall-related injuries, and improving patient satisfaction and nursing compliance.

Previous studies have emphasized that fall risk assessment is a critical first step in fall prevention (24–26). By accurately assessing a patient’s fall risk, high-risk individuals can be identified, and tailored preventive measures can be implemented to reduce fall incidence (27, 28). However, many conventional tools may not adequately capture dynamic risk factors (11). In this study, we introduced a more comprehensive and detailed fall risk assessment method. This enhanced version not only includes basic patient information but also incorporates multidimensional factors like physical activity ability, balance function, and cognitive status. Compared to traditional evaluation methods, which often rely heavily on static factors (29), this comprehensive approach allows for more precise identification of high-risk individuals, providing a scientific basis for personalized fall prevention strategies.

The formulation and implementation of personalized preventive measures significantly improve fall prevention outcomes (30–32). This study carefully considered individual patient differences and needs when designing preventive interventions. Based on the results of the improved fall risk assessment and the obstacle physical activity test, we developed customized preventive strategies for each patient. These measures were not only targeted but also well-accepted by patients, significantly improving the overall effectiveness of fall prevention. The integration of patient-specific data from dynamic tests like the obstacle course appears crucial for this personalization (33).

Environmental management plays a crucial role in fall prevention (23). Studies have shown that improving the hospital environment and enhancing safety facilities significantly reduce the risk of falls. In our research, we identified the rationality of ward layouts and the configuration of safety facilities as key factors influencing fall risk. By improving the ward layout, adding safety facilities, and enhancing education, we created a safer and more comfortable environment for patients. This not only reduced environmental risks but also improved the overall patient experience and satisfaction.

To ensure the effective implementation and continuous improvement of fall prevention measures, we established a comprehensive fall prevention and supervision mechanism. The establishment of a robust fall prevention and supervision mechanism requires attention to several factors, such as the accuracy of evaluation methods, the relevance of preventive measures, and the effectiveness of environmental management (34). Additionally, we leveraged the hospital’s standard incident reporting system for fall events to systematically gather data, analyze root causes, and inform improvements in our prevention process.

Despite the positive outcomes, this study has several limitations. First, as a single-center study, the findings may not be fully generalizable to other healthcare settings with different patient populations, staffing models, or environmental contexts. Second, the follow-up period was limited to the duration of the hospital admission. We did not assess post-discharge fall rates, which limits our understanding of the long-term effectiveness of the intervention. A significant limitation is that the assessment of Quality of Life, while systematic in its administration, did not employ a standardized, internationally validated QoL instrument. Instead, it relied on a structured, unvalidated questionnaire focusing on perceived well-being and functional status. This approach may introduce subjectivity and limit comparability with other studies. Future studies should prioritize the incorporation of such validated QoL instruments to strengthen the evidence regarding this outcome.

## 5 Conclusion

In conclusion, this study demonstrates that personalized fall prevention strategies guided by an improved fall risk assessment form combined with the obstacle physical activity test can effectively reduce the incidence of falls among older adults inpatients, reduce fall-related injuries, and improve nursing satisfaction, compliance, and quality of life. Therefore, this integrated assessment and intervention method holds great potential for broader implementation in the fall prevention care of older adults inpatients.

## Data Availability

The original contributions presented in the study are included in the article/Supplementary material, further inquiries can be directed to the corresponding author.
