# Supplementary material for: Effectiveness of an improved fall risk assessment form combined with obstacle physical activity testing in preventing falls in older adults hospitalized patients
Source: Front Public Health. 2025 Oct 28;13:1601666. doi: 10.3389/fpubh.2025.1601666 (PMC12602510; doi:10.3389/fpubh.2025.1601666)
Supplement: Supplementary file 1 [file Table_1.docx]

**Supplementary Table S1. Inpatient Fall Risk Assessment Form**

| **Assessment Item** | **Score** | | | |
| --- | --- | --- | --- | --- |
|  | **1 point** | **2 points** | **3 points** | **4 points** |
| **Age** | 65-75 years or < 5 years | > 75 years |  |  |
| **Medical History** | Has epilepsy history | History of a fall (bed fall) in the past year | History of a fall (bed fall) in the past 3 months |  |
| **Diseases** | Orthostatic hypotension, blood pressure < 90/60 mmHg | Mild anemia (Hb > 90 g/L) | Moderate anemia (Hb 60–90 g/L) | "Blood loss > 500ml, severe anemia (Hb 30-59 g/L)" |
| **Test Results** | Blood glucose, potassium, sodium, calcium within normal ranges | Critical values or extreme anemia (Hb < 30 g/L) |  |  |
| **Consciousness** | Drowsy | Confused consciousness (delusions, hallucinations, delirium, agitation) |  |  |
| **Vision** | Visual impairment |  |  |  |
| **Hearing** |  |  |  |  |
| **Balance Function** | Balance dysfunction |  |  |  |
| **Physical Activity** | Requires cane, crutch, or walker, or reports dizziness or weakness | Orientation impairment, no control of own actions | Physical disability (limb deformity, hemiplegia, unstable gait) |  |
| **Excretion** | Incontinence | Diarrhea, frequent urination |  |  |
| **Medication** | Laxatives, diuretics, antihypertensive, antidiabetic, antiepileptic, mydriatic, antidepressants, muscle relaxants, sedatives, analgesics, etc. |  |  |  |
| **Cognitive Status** | Overconfident | Mild dependence | Moderate dependence | Severe dependence |
| **Total Score** |  |  |  |  |

**Note:**

- **Total score ≥ 8** or a single item score ≥ 4 indicates a high-risk fall patient, who should be provided with a fall risk education form.
- Dynamic re-assessment is required when a patient's self-care ability or critical values change. It is recommended to assess once a week.

**Critical Values:**

- Blood glucose ≤ 2.2 mmol/L or ≥ 22.2 mmol/L
- Potassium ≤ 2.8 mmol/L or ≥ 6.2 mmol/L
- Sodium ≤ 120 mmol/L or ≥ 160 mmol/L
- Calcium < 1.6 mmol/L or > 3.5 mmol/L
- Hemoglobin ≤ 50 g/L

**Orientation Impairment:**
Loss of awareness or incorrect awareness of the environment (time, location, people) or oneself (name, age, occupation, etc.) is considered orientation impairment.

**Balance Dysfunction:**
Impairment of the vestibular system, proprioceptive system, or visual system. Mild dysfunction may present as difficulty walking, moderate dysfunction as an inability to stand, and severe dysfunction as an inability to sit or stand.

**Visual Impairment:**
Impaired vision in both eyes that cannot be corrected or a restricted field of vision affecting daily life and social participation.
