# Supplementary material for: Effectiveness of an improved fall risk assessment form combined with obstacle physical activity testing in preventing falls in older adults hospitalized patients
Source: Front Public Health. 2025 Oct 28;13:1601666. doi: 10.3389/fpubh.2025.1601666 (PMC12602510; doi:10.3389/fpubh.2025.1601666)
Supplement: Supplementary file 2 [file Table_2.docx]

**Supplementary File S2: Quality of Life Questionnaire**

**Patient ID:**

**Date of Assessment (Admission/Discharge):**

**Assessed by:**

*This questionnaire asks about your health and well-being during your hospital stay. Please answer each question based on how you have been feeling over the past week. There are no right or wrong answers.*

**Part 1: Physical Functioning & Daily Activities**

**1. Mobility:**

How much difficulty do you currently have with moving around (e.g., walking in your room, to the bathroom)?

- ( ) No difficulty
- ( ) Slight difficulty
- ( ) Moderate difficulty
- ( ) Severe difficulty
- ( ) Unable to move around at all

**2. Self-Care Activities:**

How much difficulty do you currently have with personal care activities (e.g., dressing, bathing, using the toilet)?

- ( ) No difficulty
- ( ) Slight difficulty
- ( ) Moderate difficulty
- ( ) Severe difficulty
- ( ) Completely unable to do them myself

**3. General Physical Activity:**

Compared to your usual ability before this hospital admission (or since the last assessment), how would you rate your current ability to engage in light physical activities within the hospital (e.g., sitting up, short walks if permitted)?

- ( ) Much better
- ( ) Slightly better
- ( ) About the same
- ( ) Slightly worse
- ( ) Much worse

**Part 2: Psychological Well-being**

**4. Mood:**

Over the past week, how often have you felt down, depressed, or hopeless?

- ( ) Not at all
- ( ) Several days
- ( ) More than half the days
- ( ) Nearly every day

**5. Anxiety about Falls:**

How worried are you about falling while you are in the hospital?

- ( ) Not at all worried
- ( ) Slightly worried
- ( ) Moderately worried
- ( ) Very worried
- ( ) Extremely worried

**6. Overall Well-being:**

Considering all aspects of your health, how would you rate your overall sense of well-being right now?
(Please mark on the scale, where 0 is very poor and 10 is excellent)

0 1 2 3 4 5 6 7 8 9 10

( )Very Poor Excellent( )

**Part 3: Social Interaction (within the hospital setting)**

**7. Interaction with Staff/Others:**

How satisfied are you with your ability to interact with hospital staff or other patients (if applicable and desired)?

- ( ) Very satisfied
- ( ) Satisfied
- ( ) Neutral
- ( ) Dissatisfied
- ( ) Very dissatisfied

**Part 4: Global Perceived Health & Change (Administered at Discharge/Follow-up)**

**8. Overall Health Status:**

In general, how would you say your health is now compared to when you were admitted to the hospital (or at the time of the last assessment)?

- ( ) Much better now
- ( ) Somewhat better now
- ( ) About the same
- ( ) Somewhat worse now
- ( ) Much worse now

**9. Perceived Change in Quality of Life:**

Overall, how would you rate the change in your quality of life since admission (or the last assessment)?

- ( ) Markedly improved
- ( ) Raised (somewhat improved)
- ( ) Uniformity (no change)
- ( ) Reduced (somewhat worse)
- ( ) Markedly worse

**For Assessor Use (to be completed after patient interview/assessment):**

**Overall QoL Score (if applicable, based on scoring a predefined subset of questions):**

**Categorization of QoL Change (based on overall score change and/or global perceived change Q9, corroborated by nursing assessment):**

- ( ) Improve markedly
- ( ) Raise
- ( ) Uniformity
- ( ) Reduce

**Assessor Notes (optional):**
